# Supplementary material for: Chimeric Antigen Receptor T Cell Therapy Targeting Epithelial Cell Adhesion Molecule in Gastric Cancer: Mechanisms of Tumor Resistance
Source: Cancers (Basel). 2023 Nov 23;15(23):5552. doi: 10.3390/cancers15235552 (PMC10705754; doi:10.3390/cancers15235552)
Supplement: Supplementary file 1 [file cancers-15-05552-s001.zip › Supplementary Figures.pdf]

Chimeric Antigen Receptor T Cell Therapy Targeting Epithelial Cell Adhesion Molecule in Gastric Cancer: Mechanisms of Tumor Resistance

Yanping Yang [1,2,†], Raymond Louie [3,†], Janusz Puc [4], Yogindra Vedvyas [1,2], Yago Alcaina [2], Irene M. Min [1,5], Matt Britz [4], Fabio Luciani [6,*] and Moonsoo M. Jin [1,2,5,*]

1 Department of Radiology, Houston Methodist Research Institute, Houston, TX 77030, USA; imin@houstonmethodist.org (I.M.M.)
2 Molecular Imaging Innovations Institute, Department of Radiology, Weill Cornell Medicine, New York, NY 10065, USA; yalcaina@ratiotx.com
3 School of Computer Science and Engineering, University of New South Wales (UNSW), Sydney, NSW 2052, Australia; r.louie@unsw.edu.au
4 AffyImmune Therapeutics, Inc., Natick, MA 01760, USA
5 Department of Surgery, Weill Cornell Medicine, New York, NY 10065, USA
6 School of Medical Sciences and Kirby Institute for Infection and Immunity, University of New South Wales (UNSW), Sydney, NSW 2052, Australia
* Correspondence: luciani@unsw.edu.au (F.L.); mjin@houstonmethodist.org (M.M.J.)
† These authors contributed equally to this work.

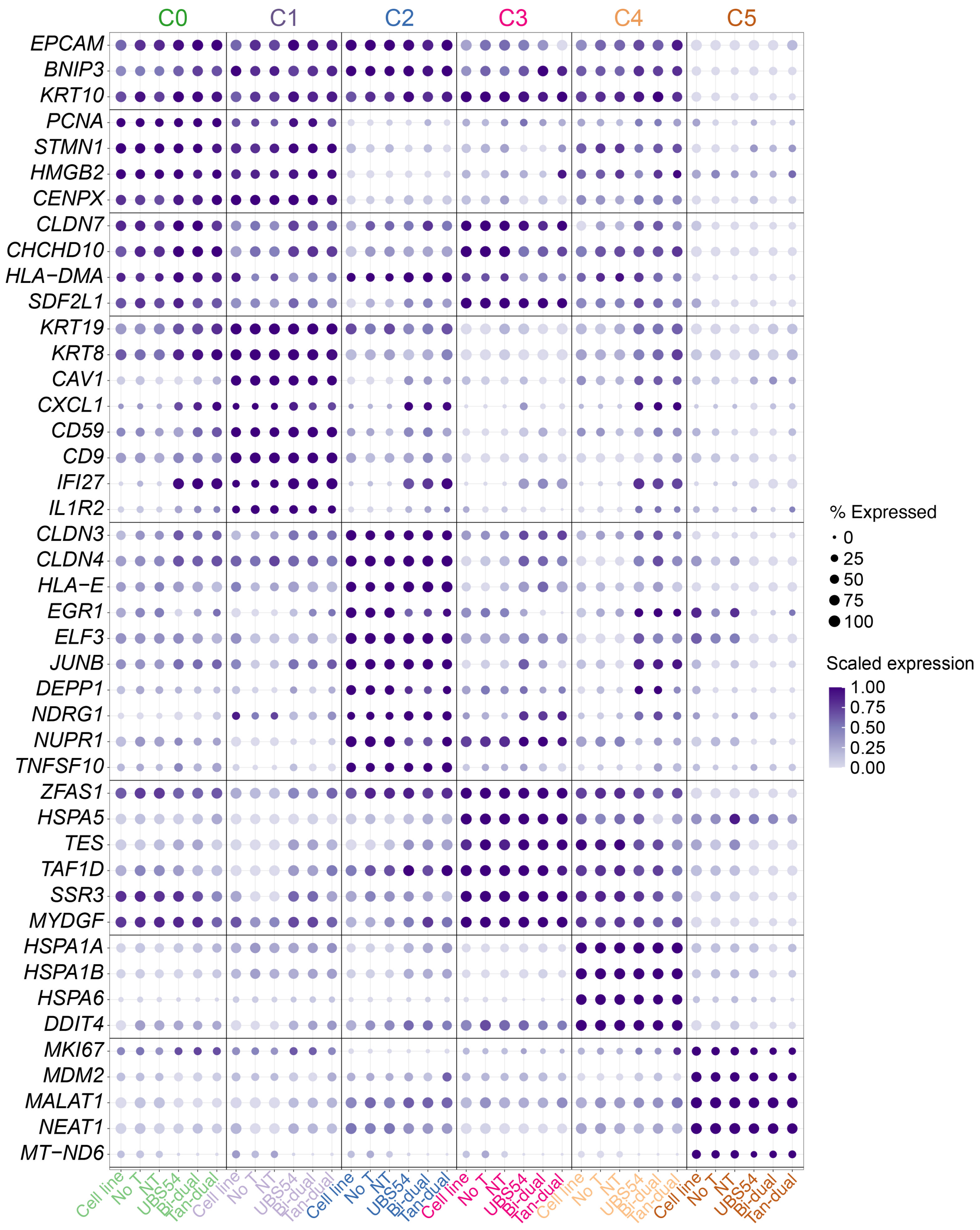

Figure S1. Dot plots representing cluster genes measured as average gene expression normalized by sample of origin.

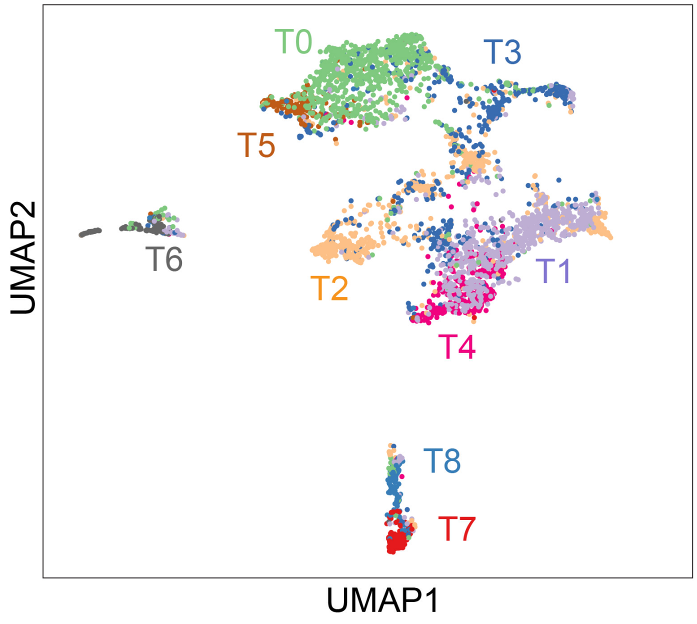

Figure S2. UMAP based on the scRNA-seq analysis of T cells after regression of cell cycle.

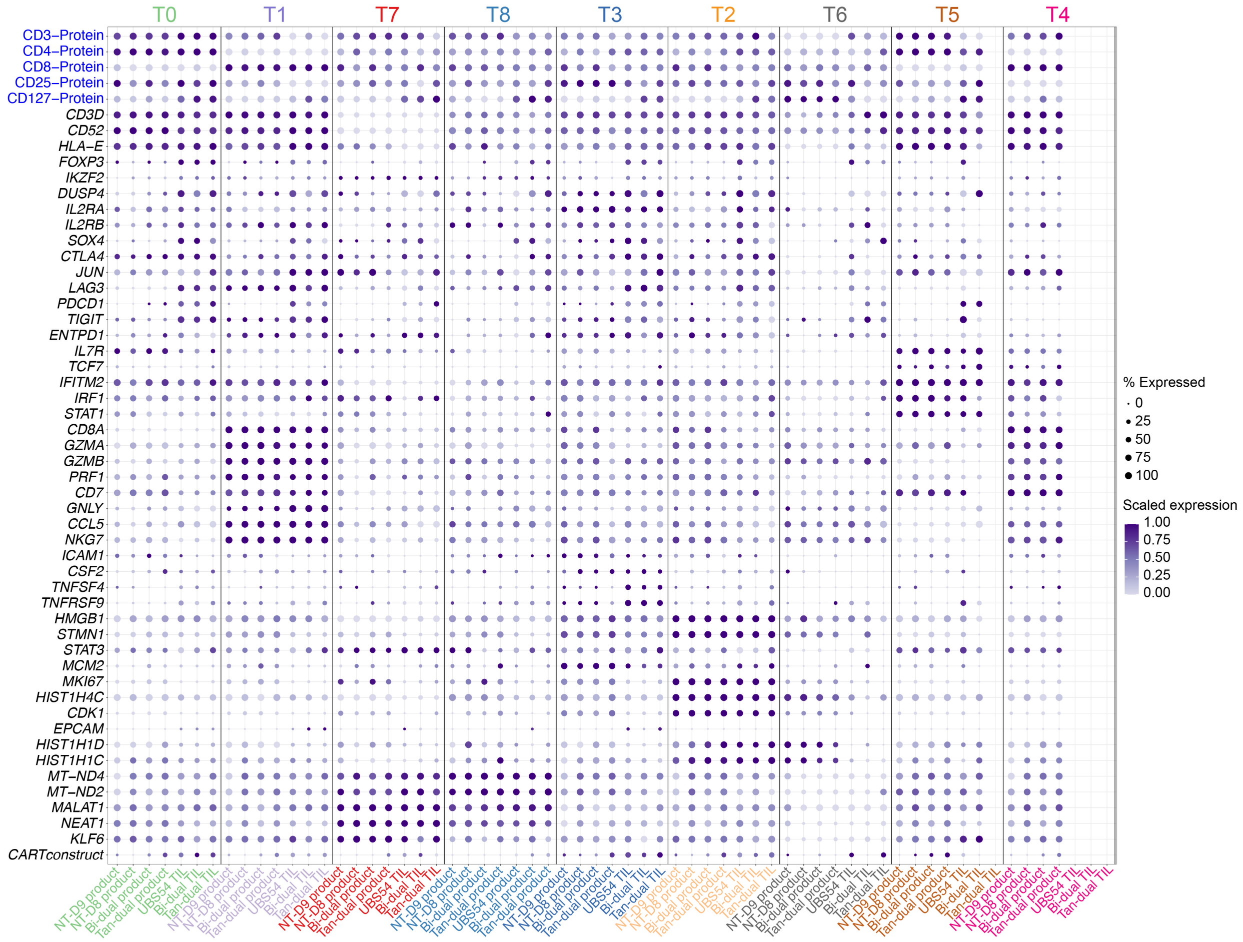

Figure S3. Dot plot representing cluster genes for each cluster. Each column represent a sample.

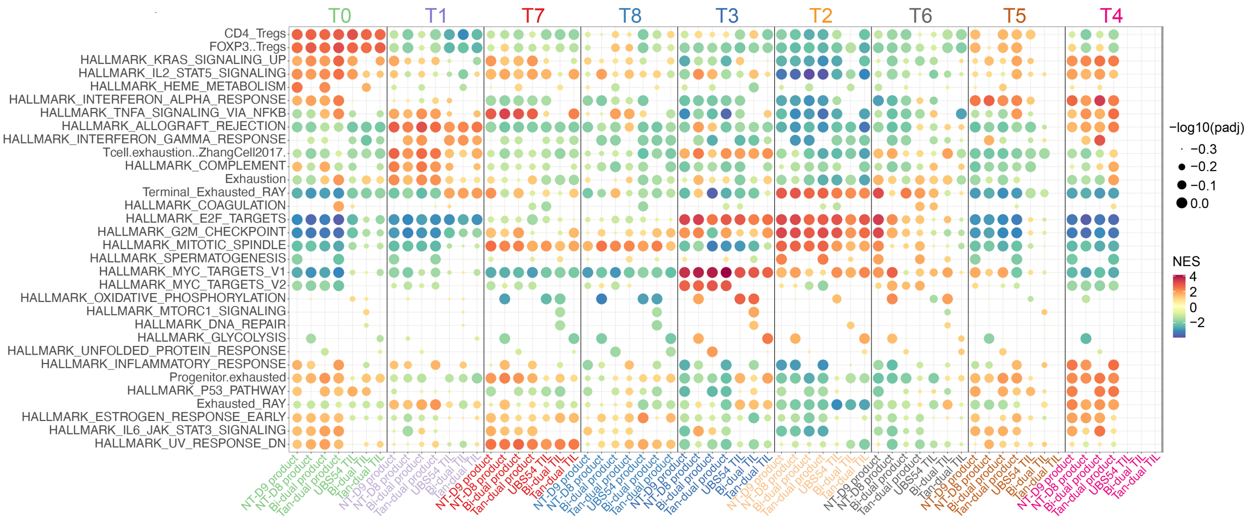

Figure S4. Dot plot representing GSEA for cluster genes. Each column represent a sample.
